# RNAProbe: a web server for normalization and analysis of RNA structure probing data

**DOI:** 10.1093/nar/gkaa396

**Published:** 2020-06-06

**Authors:** Tomasz K Wirecki, Katarzyna Merdas, Agata Bernat, Michał J Boniecki, Janusz M Bujnicki, Filip Stefaniak

**Affiliations:** Laboratory of Bioinformatics and Protein Engineering, International Institute of Molecular and Cell Biology in Warsaw, ul. Ks. Trojdena 4, PL-02-109 Warsaw, Poland; Laboratory of Bioinformatics and Protein Engineering, International Institute of Molecular and Cell Biology in Warsaw, ul. Ks. Trojdena 4, PL-02-109 Warsaw, Poland; Laboratory of Bioinformatics and Protein Engineering, International Institute of Molecular and Cell Biology in Warsaw, ul. Ks. Trojdena 4, PL-02-109 Warsaw, Poland; Laboratory of Bioinformatics and Protein Engineering, International Institute of Molecular and Cell Biology in Warsaw, ul. Ks. Trojdena 4, PL-02-109 Warsaw, Poland; Laboratory of Bioinformatics and Protein Engineering, International Institute of Molecular and Cell Biology in Warsaw, ul. Ks. Trojdena 4, PL-02-109 Warsaw, Poland; Institute of Molecular Biology and Biotechnology, Faculty of Biology, Adam Mickiewicz University, ul. Umultowska 89, PL-61-614 Poznan, Poland; Laboratory of Bioinformatics and Protein Engineering, International Institute of Molecular and Cell Biology in Warsaw, ul. Ks. Trojdena 4, PL-02-109 Warsaw, Poland

## Abstract

RNA molecules play key roles in all living cells. Knowledge of the structural characteristics of RNA molecules allows for a better understanding of the mechanisms of their action. RNA chemical probing allows us to study the susceptibility of nucleotides to chemical modification, and the information obtained can be used to guide secondary structure prediction. These experimental results can be analyzed using various computational tools, which, however, requires additional, tedious steps (e.g., further normalization of the reactivities and visualization of the results), for which there are no fully automated methods. Here, we introduce RNAProbe, a web server that facilitates normalization, analysis, and visualization of the low-pass SHAPE, DMS and CMCT probing results with the modification sites detected by capillary electrophoresis. RNAProbe automatically analyzes chemical probing output data and turns tedious manual work into a one-minute assignment. RNAProbe performs normalization based on a well-established protocol, utilizes recognized secondary structure prediction methods, and generates high-quality images with structure representations and reactivity heatmaps. It summarizes the results in the form of a spreadsheet, which can be used for comparative analyses between experiments. Results of predictions with normalized reactivities are also collected in text files, providing interoperability with bioinformatics workflows. RNAProbe is available at https://rnaprobe.genesilico.pl.

## INTRODUCTION

RNA is a highly flexible and dynamic biopolymer capable of adopting complex, intricate structures ultimately dictated by its sequence. It plays fundamental roles and regulates all steps of gene expression (1–4). To comprehend the RNA mechanism of action, including the dynamics of interactions with its ligands or protein partners, researchers need to investigate RNA structures. Biochemical approaches represent a complement to biophysical techniques, such as X-ray crystallography and NMR, by increasing the experimental range and throughput, and also allowing for RNA study in the cellular context (5). Chemical probing reveals nucleic acid conformation in solution, and depending on the type of the used reagent, it detects structural features such as base pairing, solvent accessibility and flexibility of the nucleotide chain at the single-nucleotide resolution (6). RNA treated with the reactive probe yields covalent adducts at susceptible nucleotides that can be subsequently mapped back to their position within the RNA sequence. Depending on the reagent of choice and its specificity to the RNA, the investigator can obtain information about distinct structural features.

DMS (dimethyl sulfate) and CMCT (*N*-cyclohexyl-*N*-(2-morpholinoethyl)carbodiimide methyl-*p*-toluenesulfonate) are commonly used chemicals that react with the nitrogen base moiety of RNA in a sequence-specific manner. Both compounds modify the Watson–Crick edge of nucleotides that are free from the Watson-Crick base pairing (7). DMS methylates adenine at the N1 position and cytosine at the N3 position (6), while CMCT is used mostly for modification of uracil in position N3, and can also react with the nitrogen in the N3 position of guanine (8). These modifications cause premature termination of the cDNA synthesis by the reverse transcriptase and resulting stop sites can be detected in the primer extension reaction. Because DMS and CMCT modify only single-stranded RNA, double-stranded regions are inferred by the lack of modification. In addition, DMS modifies guanine at the N7 position of the Hoogsten edge, which does not create reverse transcriptase stops, but can be detected by cleavage of the modified RNA after borohydride reduction and aniline cleavage (7).

SHAPE (selective 2′-hydroxyl acylation analyzed by primer extension, (9,10)) is a more recent technique regarded as the gold standard of a chemical probing strategy (11), developed to study the accessibility and flexibility of RNA backbone. RNA-modifying agents applied in this method, e.g., most commonly used in *in vitro* experiments NMIA (*N*-methyl isatoic anhydride), or applied in RNA probing *in vivo* 1M7 (1-methyl-7-nitroisatoic anhydride) (9,10), are sequence-unbiased. They form an adduct with the 2′-hydroxyl group of ribose of nucleotides found in the flexible or disordered regions and discriminate them from rigid residues, e.g., involved in base pairing in double-stranded, helical regions (9,10).

Regardless of the type of modifying agent used in the experiment, classical chemical probing begins with the RNA folding, followed by chemical modification with structural probes, and detection of modification sites in the reverse transcription reaction. Finally, the read-out, previously by denaturing PAGE, and more recently superseded by capillary electrophoresis (CE), is performed in order to map modification positions along the RNA sequence. The resulting CE chromatograms can be subsequently analyzed using diverse software tools, such as QuShape (12), to obtain quantitative reactivity information from the longest readable region of the studied RNA. QuShape was designed to perform analyses of probing data in general, and at first demonstrated to work with the SHAPE experimental data (12), but it was also applied to analyze the reactivity patterns obtained with DMS or CMCT probing (13,14).

Many tools for RNA structure prediction have been adapted to use chemical probing data, however only a few of them, e.g., RNAfold from ViennaRNA (15), and Fold and ShapeKnots from RNAstructure (16) use the original reactivity patterns as an input rather than interpreted data such as predictions whether each of the residues is paired or unpaired. The use of the experimental data is, however, not straightforward, as the data requires pre-processing to serve as an input. Proper normalization of probing results and integration of multiple online tools applied for data processing and visualization poses a technical challenge. Performing the analyses in a unified way and comparing results between consecutive experiments requires following a strict normalization protocol, some experience with bioinformatic software, and a significant amount of time spent on each analysis.

Various computational tools have been developed for RNA structure prediction based on the chemical probing data, e.g., RNAex (17), RNA Framework (18) or StructureFold (19). However, these tools focus on high-throughput probing experiments (usually at the level of the whole transcriptomes) analyzed with next-generation sequencing. Increasing popularity of RNA secondary structure analyses across whole transcriptomes has prompted the development of several databases serving as repositories for high-throughput probing data from various types of experiments, e.g., Structure Surfer (20), FoldAtlas (21) and RSVdb (22). However, these databases do not allow the user to analyze their own experimental data on the fly. Instead, they provide results of previous experiments, mostly done in a high-throughput fashion for entire transcriptomes of model organisms.

To date, there has been no fully automated and simple tool dedicated to processing RNA chemical probing data from experiments focused on studying individual RNA molecules and analyzed with capillary electrophoresis. A few components of the workflow, which may facilitate the processing of the experimental data, are available. Macro_CE (https://github.com/afafbioinfo/Macro_CE) is a standalone Python script for processing QuShape data with implemented normalization protocol. IPANEMAP (https://github.com/afafbioinfo/IPANEMAP) is a set of Python scripts for predicting RNA secondary structures based on various chemical probing data (including SHAPE and DMS). It computes and outputs one or several secondary structures, corresponding to the conformers best supported by experimental data and thermodynamics. All these tools and programs may be useful at various stages of the RNA chemical probing data analysis, but they need to be installed and run locally from the command line, which is often a limiting factor for the inexperienced user.

We have developed RNAProbe—a web server for normalization and analysis of RNA chemical probing data from low-throughput experiments focused on individual RNA molecules. RNAProbe is designed to automate the analysis protocol and to integrate various tools in one place. It performs normalization of SHAPE, CMCT, and DMS probing reactivities (9,10), and applies the data to predict and visualize RNA secondary structure. The application of the server significantly decreases the time required to perform a complete analysis to a couple of minutes, in contrast to a cumbersome procedure that the researcher must perform by hand. Images generated by the server are of publication-quality, and in each analysis, the normalization and image generation is conducted precisely the exact same way, ensuring the consistency of analyses performed for a series of experiments.

## MATERIALS AND METHODS

### Workflow overview

**Figure 1.**
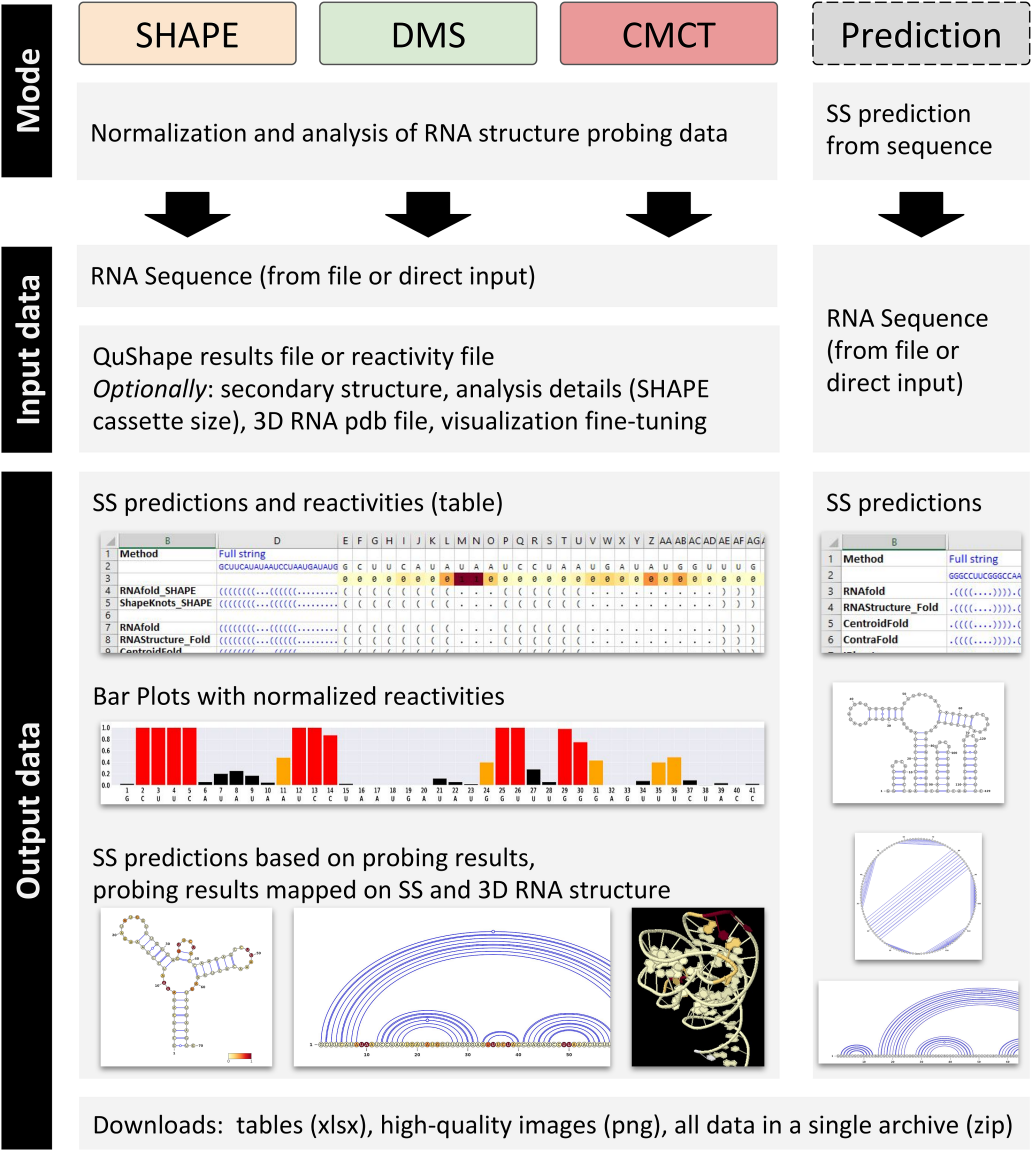
Modes of operation of the RNAProbe server (‘SHAPE’, ‘DMS’, and ‘CMCT’ modes for processing of the probing results, and the ‘Prediction mode’ for secondary structure prediction based solely on the sequence), input data required for analysis and output generated.

The purpose of RNAProbe is to facilitate and streamline the analysis of RNA chemical probing results. The server performs normalization of probing reactivities according to the standard normalization protocol for SHAPE (23), utilizes probing-directed or sequence-only secondary structure prediction methods, and finally visualizes the resulting structures in publication-quality representations (Figure 1). The server has four modes of analysis: ‘SHAPE’, ‘DMS’, ‘CMCT’ and ‘Prediction’. In the first three modes, the analysis can be performed either for the ‘raw’ (non-normalized) reactivities or for user-provided pre-normalized reactivities. The ‘Prediction mode’ executes secondary structure predictions, based solely on the RNA sequence. For the probing modes, the server delivers VARNA representations of the RNA secondary structure (24) with applied normalized reactivity values. All predictions and reactivities are also presented as an MS Excel spreadsheet, and are accompanied by a set of plain text files with predictions in dot-bracket notation. Additionally, probing results can also be mapped on the secondary and/or 3D structure provided by the user. In the ‘Prediction mode’, the web server generates VARNA representations of the secondary structure consensus calculated based on series of secondary structure predictions obtained from various methods, a table in the Excel format with these predictions, and plain text files with dot-bracket structures.

### Implementation details

#### Normalization protocol

After analyzing the probing results with QuShape, one must perform an additional normalization of the nucleotide reactivities. RNAProbe server does it automatically for the input data provided by the user. In order to remove outliers, the server follows the protocol described previously for the SHAPE reaction (23). Briefly, from all reactivity values, 10% of the most reactive peaks are selected, and 20% of them are excluded. Then, from the remaining 8% of most reactive positions, the mean value is calculated, and all intensities are divided by it, yielding the normalized values. Finally, all reactivities with values larger than 1 are set to 1, while negative reactivities are set to 0. The whole process results in the normalized probing reactivity ranging from 0 to 1. Additionally, all nucleotides within the probed sequence that are lacking the readout are assigned with the reactivity -1. The user can also use an option of DMS/CMCT specific normalization of reactivity values, where RNAProbe uses only reactivities reported for the residues expected to react with the chemical probe (C and A for DMS, G and U for CMCT) instead of using all reactivities (as in SHAPE normalization).

#### Structure prediction methods

For probing-guided secondary structure prediction, RNAProbe makes use of three methods. For SHAPE-guided predictions, the methods used for calculating secondary structures are: ViennaRNA RNAfold (RNA probing mode) (15), and RNAstructure ShapeKnots (16). For DMS and CMCT modes, probing-guided predictions are obtained using RNAstructure Fold (16). The reason for applying different methods for SHAPE and for DMS/CMCT analyses is due to the limitations of computational methods that utilize the relative reactivities in the process of structure prediction. RNAfold has a dedicated algorithm for processing SHAPE reactivities. ShapeKnots is another computational method dedicated for processing SHAPE reactivities, with the ability to predict pseudoknots. Additionally, ShapeKnots has an algorithm for DMS-probing data. Lastly, Fold is the most universal computational method as it has algorithms for processing all three types of probing reactivities mentioned above. However, RNAProbe server uses Fold only for processing DMS/CMCT data, since ShapeKnots, which supersedes Fold in the ability to predict pseudoknots, is already applied for processing SHAPE data. SHAPE-directed RNAfold uses reactivities converted to pseudo-energy contributions for every nucleotide in a stacked pair, obtained by a linear equation (25). ShapeKnots applies reactivity information to generate an additional pseudo-free energy change term for the folding algorithm (26). RNAstructure Fold DMS/CMCT-guided predictions are obtained by transforming the probing reactivities to pseudo-energies, and by adding a reward or a penalty, depending on whether paired nucleotides were modified by the probe (27). We decided not to implement the conversion of experimental reactivities to a simplified binary classification paired vs. unpaired to be used as folding constraints, as this could introduce unnecessary biases at the early stage of the prediction.

For sequence-only predictions, RNAProbe makes use of ViennaRNA RNAfold (15), RNAstructure Fold and ProbKnot (16), CentroidFold (28), CONTRAFold (29), IPknot (30), LinearFold-V and LinearFold-C (31). For calculating a consensus structure from a series of predictions, RNAProbe uses an in-house method, which identifies base pairs that are present in at least 50% of the input predictions. All structure prediction methods are used with their default settings, as in their original stand-alone or web server implementations.

### Inputs

RNA sequence is required in all analysis modes. It can be uploaded either as a text file containing the RNA sequence or pasted into the ‘direct input’ window. The input sequence must be the same as it was used in the QuShape analysis (including the cassette); the sequence to be analyzed can be specified by selecting a region of interest (ROI), leaving out the cassette on 5′ and 3′ ends of the RNA molecule. The sequence should consist of only four letters corresponding to the four RNA nucleotides - A, C, G, U (either capital or lower case); should T be present in the sequence, it will be displayed as U in the output files.

The QuShape file is a tab-separated text file, generated by the QuShape software (12). The sequence is read from the ‘seqRNA’ column and compared to the input sequence file. If the server detects any inconsistencies between the two, it will report them to the user. The user can also choose the number of nucleotides for both 5′ and 3′ ends that will not be analyzed, e.g., the user does not want to have SHAPE cassette being considered during predictions. The user can upload several QuShape files in one analysis (e.g., to calculate the average value from experimental replicates for the same sequence), and the reactivities will be normalized before the averaging. The reactivities file can be used instead of the QuShape file. It is a text file containing two space-separated columns: the nucleotide numbers and the corresponding normalized reactivity values. For nucleotides or regions with unknown reactivity, this value will be set to –999. The file format is the same as in SHAPE-directed predictions with RNAfold and ShapeKnots. The numbering must be consistent with the nucleotide position within the sequence. This option is intended to allow the user to visualize/analyze the data that was normalized following a protocol other than the one the present web server applies.

The secondary structure input is optional. This option allows for mapping reactivities onto the provided structure (instead of or in addition to probing-based predictions generated by RNAProbe). The secondary structure in a dot-bracket notation can be uploaded either as a text file or pasted directly into the ‘direct input’ box. The 3D structure input is also optional. It allows for mapping of reactivty values on the provided PDB structure, to obtain color-coded visualization of the probing intensties on the 3D model.

### Outputs

The RNAProbe server generates high-quality images, most of which are displayed on the results page. These include: a table with secondary structure predictions in dot-bracket format, a heatmap and a barplot displaying the reactivity profile of the molecule under study (not available in ‘Prediction mode’), and VARNA representations of the predicted secondary structures. The non-displayed results include a text file containing predictions in dot-bracket format and a normalized reactivities file. All the generated outputs can be downloaded separately, or as a zip archive.

#### XLSX/HTML table

The table with all results is available in two formats: HTML viewed on the website, and XLSX file, suitable for download. The table is divided into five main parts: Name—the job name provided by the user; Method—lists the methods used to predict the secondary structure(s); Filename—a combined name of the uploaded file, along with the analysis mode; Full string—sequence/secondary structure, represented as one string of characters; Sequence/reactivity/structure—information for each nucleotide placed in a separate cell ( type of nucleotide, reactivity value, structure in dot-bracket notation).

#### VARNA representations for predicted structures

The predicted or user-defined structures are represented using VARNA (24). For each analysis mode, the structures are generated in three different VARNA representations: radiate—the default representation; circular—the structure is represented in a circular form with lines connecting paired nucleotides; and, linear - the structure is represented as a straight line, with base pairs connections represented as arches. In ‘SHAPE mode’, the generated/displayed secondary structures are SHAPE-directed predictions using RNAfold (15) and ShapeKnots (16), color-coded as to illustrate the range of reactivity values. In ‘DMS’ and ‘CMCT’ modes, generated structures are a result of the probing-directed Fold method (16). The user can choose to color only the nucleotides that are susceptible to the given reagent, and to ‘grey-out’ the nucleotides that are not supposed to be modified. In the ‘Prediction mode’, the structures are visualized without any color code.

#### Mapping of reactivities on the 3D structure

If the user supplies an RNA 3D structure in the PDB format, the probing data can be mapped onto the B-factor fields of the respective residues. RNAProbe visualizes the mapping using JSmol (32), additionally, the user can download the modified output PDB file and display the structure, using any molecular structure viewer that has a capability to color the 3D structure according to the B-factor value.

#### Heatmap and barplot

The heatmap and barplot images are available for all three probing modes. The heatmap plot presents the reactivity value for each nucleotide in a separate box according to a color-range, either a default one for each probing method (SHAPE—yellow-orange-red, DMS—yellow-green, CMCT—white-red) or a defined one by the user from a set of available alternative coloring schemes. The barplot represents the reactivity value for each nucleotide, where the reactivities are colored as follows: 0.0 to 0.3—black, 0.3 to 0.7—orange, 0.7 to 1.0—red. With the heatmap and barplot representations, the user can easily spot differences in reactivities of different residues of a particular RNA sequence, as well as compare the reactivity profiles resulting from different experiments.

#### Text files with predictions

The server generates two text files with results, providing interoperability with other bioinformatic workflows. All predictions file consists of the sequence and the predicted secondary structures for all applied prediction methods. The reactivities file is a text file with two columns—a nucleotide number (reflecting its position in the sequence) and its corresponding reactivity value. The format of this file is the same as the one used in SHAPE-directed predictions with RNAfold and ShapeKnots. Note that this file is not available in ‘Prediction mode’.

### Experimental setup

The *add* riboswitch aptamer domain is an allosterically regulated element, found in the 5′ UTR of the adenosine deaminase mRNA of the Gram-negative bacterium *Vibrio vulnificus*, and it regulates the translation of a downstream gene in response to adenine *in vivo* (33). Its structure and ligand binding mechanism has been studied and determined by a wide range of biochemical and biophysical techniques, including SHAPE probing (34), and X-ray crystallography (35), and also in combination with computational methods (36 ). In the analysis conducted for the purpose of illustrating the functionality of RNAProbe, the sequence of this riboswitch was combined with the SHAPE cassette as described by Wilkinson *et al.* (10). Transcripts were produced by *in vitro* transcription with T7 RNA polymerase. All RNA transcripts were purified by PAGE under denaturing conditions.

SHAPE probing was performed following an established protocol (10). Shortly, each reaction (positive, negative, or sequencing) required 2 pmol of the RNA construct. The RNA was resuspended in 0.5× TE buffer (5 mM Tris pH 8.0, 0.5 mM EDTA), denatured at 95°C for 2 min, and plunged on ice. The samples were then mixed with 3× SHAPE folding buffer (333 mM HEPES pH 8.0, 20 mM MgCl_2_ 333 mM NaCl) and refolded at 37°C for 15 min. When the chemical probing was carried out in the presence of a ligand, adenine was added to the RNA sample at 1 mM concentration in the 3× folding buffer. RNA modification, initiated by the addition of 130 mM NMIA dissolved in DMSO, was carried out at 37°C for 45 min. Since NMIA undergoes hydrolysis, the reaction was self-quenching. The negative control was treated with the same volume of DMSO. After incubation with the probing reagent, RNA was precipitated in the presence of ethanol. Resulting pellets were resuspended in 0.5× TE buffer, and combined with fluorescent primers for the reverse transcription with the SuperScript III Reverse transcriptase kit, according to the manufacturer's instructions (different fluorophores are required for the positive/negative reactions and for the sequencing reaction). In addition to the standard reaction mix, sequencing reactions also contained the ddNTP of choice. Resulting cDNA samples were precipitated in ethanol, and recovered pellets were resuspended in Hi-Di formamide. After combining cDNA from positive/negative reactions with the sequencing ladder, samples were resolved by capillary electrophoresis, and the signal alignment to the corresponding sequence was performed in QuShape.

DMS and CMCT probing experiments, in contrast to the SHAPE experiments, were performed only for the adenine riboswitch RNA in the absence of the ligand. The previously described protocol for DMS probing (37) was optimized as follows: 2.5 pmol of RNA, required for a single reaction, were dissolved in 0.5x TE buffer. RNA denaturation was carried out in the same conditions as for SHAPE probing. 2.5× DMS folding buffer (750 mM sodium cacodylate, 25 mM MgCl_2_) was added to the RNA, the mix was incubated at 37°C for 15 min. Then, the RNA was treated with DMS dissolved in ethanol and incubated at room temperature for 10 min. At the same time, the negative control was treated with ethanol only. The reaction was quenched by the addition of 14.7 M β-mercaptoethanol and precipitated with ethanol.

The CMCT probing protocol was adapted from (38). 1 pmol of RNA, required for a single reaction, was dissolved in 3.5 μl of water supplemented with 111 mM KCl and 89 mM K-borate. The RNA was denatured at 95°C for 2 min and chilled on ice for 1 min. RNA samples were mixed with MgCl_2_ to achieve a final concentration of 100 mM and RNA folding was completed by incubating RNA at 37°C for 15 min. RNA was then treated with 300 mM CMCT, while negative controls were mixed with water. Both kinds of samples were incubated at room temperature for 20 min. The modification was quenched simply by ethanol precipitation. For both DMS and CMCT probing experiments, sequencing reactions were prepared in an analogous manner as described for SHAPE probing. The RNA samples recovered after modification were further processed following the SHAPE probing protocol.

## RESULTS

### RNAProbe web server

The RNAProbe is a web server for automated normalization and analysis of RNA chemical probing results, and for probing-assisted secondary structure prediction with several computational tools. RNAProbe displays secondary structure predictions generated with and without experimental data. The analysis performed by the server is much faster than manual analysis, moreover, the results are visualized in an integrated manner. The server allows analyzing data from three different chemical probing experiments: SHAPE, DMS and CMCT probing. RNAProbe is also capable of predicting secondary structure based only on the sequence employing various secondary structure prediction tools. The server offers a convenient way of forwarding the RNA sequence with the predicted secondary structures to SimRNAweb, an RNA 3D structure prediction server (39). RNAProbe has been operating since May 2019, and so far, it has processed several hundreds of datasets.

### Example applications

To demonstrate the performance of the RNAProbe server, we chose the *add* riboswitch aptamer domain from *Vibrio vulnificus*, for which a wealth of experimental data is already available. SHAPE probing was performed either in the presence or in the absence of the natural ligand (adenine). DMS and CMCT probing analyses were performed in the absence of the ligand. Experiments were conducted in triplicates for independently prepared RNA samples. The experimental protocols are described in the ‘Materials and Methods’ section of this manuscript.

**Figure 2.**
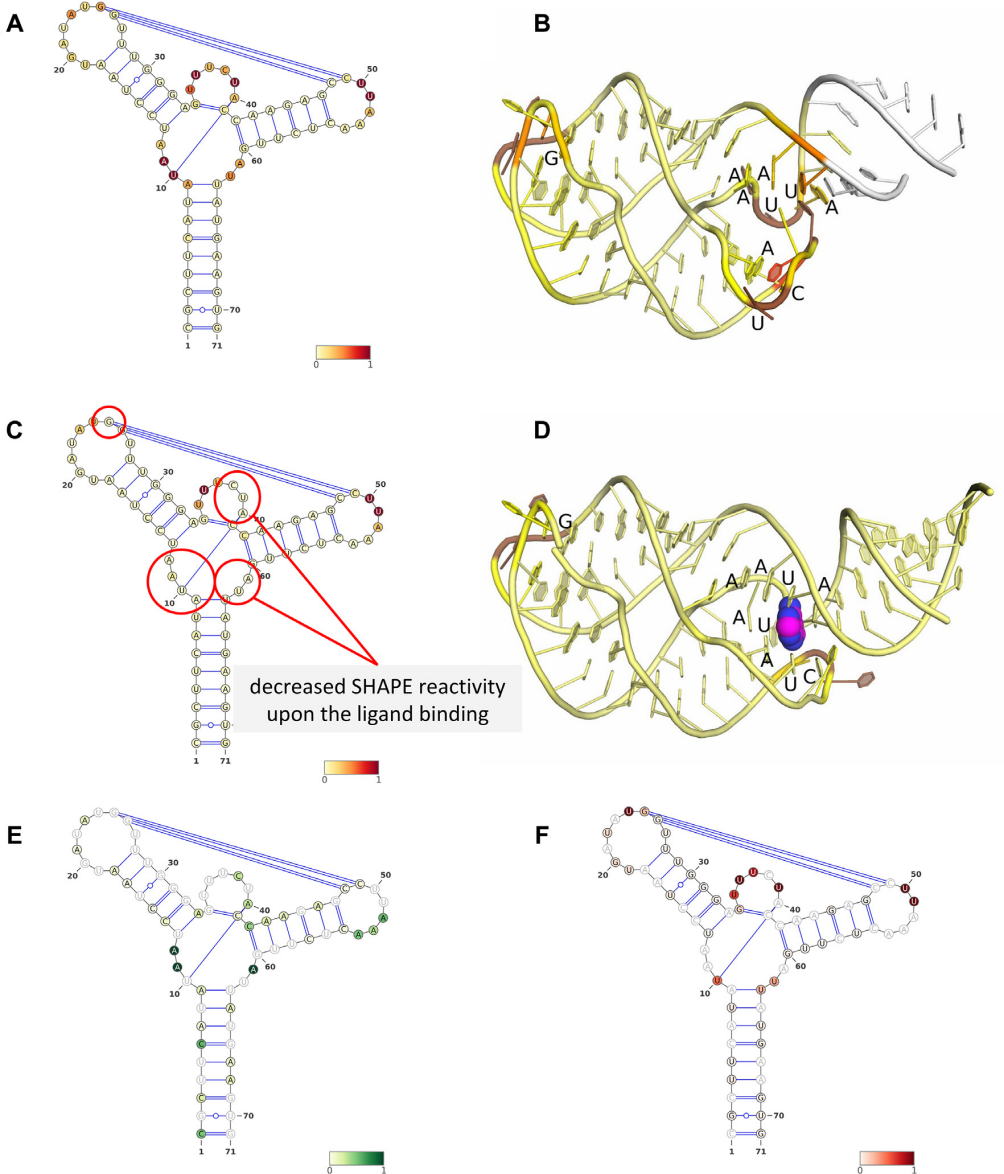
Results of chemical probing experiments obtained for the *add* riboswitch aptamer domain analyzed and visualized using the RNAProbe server. Visualization of the SHAPE probing of the riboswitch in the absence of the ligand (PDB code: 5e54 (40)) mapped on the secondary (**A**) and tertiary structure (**B**), compared to the mapping in the presence of the ligand - adenine (PDB code: 1y26 (35); ligand is presented as spheres (C—magenta, N—blue)) (**C** and **D**). The ligand-free form of the riboswitch probed with DMS (**E**) and CMCT (**F**).

Figure 2 shows how the RNAProbe server visualizes the mapping of the probing data onto the secondary structure of the *add* riboswitch aptamer domain inferred from the crystal structure (35), consisting of three helices (P1, P2 and P3), with an additional pairing that forms a pseudoknot. The reactivties are also mapped on the 3D model of the aptamer. The reactive residues of the ligand-free form map onto regions indicated as single-stranded. In the ligand-bound form, additional residues become protected, from the chemical modification, highlighting the ligand-bound site. Secondary structure prediction (regardless of the use of the experimental data) has generated a similar secondary structure pattern, albeit without the base pairs between the P2 and P3 loops, and hence without a pseudoknot.

## DISCUSSION

RNAProbe is a web server that implements an integrated RNA chemical probing data processing workflow with an intuitive interface and convenient visualization of the analysis results. Currently, the server allows for processing chemical probing data obtained with three methods - SHAPE, DMS and CMCT. Results are visualized with various plots and tables. The workflow implemented in the server reduces complicated and time-consuming analysis of the probing results to a few clicks. Additionally, RNAProbe can predict RNA secondary structure based purely on the RNA sequence, with several different prediction methods. However, RNAProbe has some limitations. The sequence length of the analyzed RNA cannot exceed 600 nucleotides. One must keep in mind that with the increasing sequence length, the prediction tools employed in the workflow become less accurate. Another limitation is that even with high-quality input data, the secondary structure prediction may be inaccurate. Having a consensus output from the different secondary structure prediction tools may indicate a higher degree of confidence for a given predicted secondary structure, yet these predictions still have to be interpreted with caution.

In future versions of the RNAProbe, we plan to extend the functionality of the server by adding protocols for processing other chemical probing data, e.g., EDC, or glyoxal probing. One of the potential new functionalities is to allow for the simultaneous analyses of data from different probing methods (e.g. using reactivity profiles of complementary methods like DMS and CMCT), which would yield not only more accurate secondary structure predictions but also decrease the time required for data analysis.

## DATA AVAILABILITY

The web server is available at https://rnaprobe.genesilico.pl. This website is free and open to all users and there is no login required.
